# Characterisation of a diazinon-metabolising glutathione *S*-transferase in the silkworm *Bombyx mori* by X-ray crystallography and genome editing analysis

**DOI:** 10.1038/s41598-018-35207-8

**Published:** 2018-11-15

**Authors:** Kohji Yamamoto, Akifumi Higashiura, Aiko Hirowatari, Naotaka Yamada, Takuya Tsubota, Hideki Sezutsu, Atsushi Nakagawa

**Affiliations:** 1 Department of Bioscience and Biotechnology, Kyushu University Graduate School, 744 Motooka, Nishi-ku, Fukuoka, 819-0395 Japan; 2 Institute for Protein Research, Osaka University, 3-2 Yamadaoka, Suita, Osaka, 565-0871 Japan; 3 Transgenic Silkworm Research Unit, Institute of Agrobiological Sciences, National Agriculture and Food Research Organization, 1-2 Owashi, Tsukuba, Ibaraki 305-8634 Japan; 4 Department of Virology, Graduate School of Biomedical and Health Sciences, Hiroshima University, 1-2-3 Kasumi, Minami-ku, Hiroshima 734-8551 Japan

## Abstract

Previously, we found an unclassified glutathione *S*-transferase 2 (bmGSTu2) in the silkworm *Bombyx mori* that conjugates glutathione to 1-chloro-2,4-dinitrobenzene and also metabolises diazinon, an organophosphate insecticide. Here, we provide a structural and genome-editing characterisation of the diazinon-metabolising glutathione *S*-transferase in *B*. *mori*. The structure of bmGSTu2 was determined at 1.68 Å by X-ray crystallography. Mutation of putative amino acid residues in the substrate-binding site showed that Pro13, Tyr107, Ile118, Phe119, and Phe211 are crucial for enzymatic function. *bmGSTu2* gene disruption resulted in a decrease in median lethal dose values to an organophosphate insecticide and a decrease in acetylcholine levels in silkworms. Taken together, these results indicate that bmGSTu2 could metabolise an organophosphate insecticide. Thus, this study provides insights into the physiological role of bmGSTu2 in silkworms, detoxification of organophosphate insecticides, and drug targets for the development of a novel insecticide.

## Introduction

Glutathione (GSH) *S*-transferases (GSTs, EC 2.5.1.18) are cytosolic enzymes that are present in both prokaryotes and eukaryotes^1^. Seven GSTs are classified in mammals, alpha, mu, pi, omega, sigma, theta, and zeta, and can be distinguished based on their amino acid sequences. Sequence identity in one class covers approximately 50% and less than 30% distributed between the other classes^2,3^. Six GST classes (delta, epsilon, omega, sigma, theta, and zeta) have been reported in dipteran insects, including *Anopheles gambiae*^4^ and *Drosophila melanogaster*^5,6^. GSH conjugation is essential for the detoxification of xenobiotics^7,8^. GSTs for insects can influence their sensitivity in insecticides^4,9^, and as the Lepidoptera comprises major agricultural pests, it is important to study lepidopteran GSTs. We have characterised diverse GSTs (delta, epsilon, omega, sigma, theta, zeta, and an unclassified GST) in the silkworm *Bombyx mori*, a lepidopteran model animal^10–16^; a sigma-class GST in the fall webworm *Hyphantria cunea*, one of the most serious lepidopteran pests of broad-leaved trees^13^; and a delta-class GST in *Nilaparvata lugens*, a rice crop pest^17^. Previously, we identified a novel GST obtained from *B*. *mori* (bmGSTu2)^18^.

In the present paper, we provide a structural and genome-editing characterisation of a diazinon-metabolising glutathione *S*-transferase in *B*. *mori*. Moreover, the crystal structure of bmGSTu2 as well as *bmGSTu2* gene disruption analysis helps clarify xenobiotic agents affect insects and contributes to a more detailed understanding of the GST system.

## Results

### X-ray structural analysis of bmGSTu2

We already overexpressed recombinant bmGSTu2 in bacteria, and purified it^18^. The purified protein was crystallised in a space group of *P*4_1_ with unit cell dimensions *a* = *b* = 86.26 Å and *c* = 58.77 Å. The structure was solved and the phases were refined; Table 1 includes relevant data. The final *R*_work_ and *R*_free_ factors were 18.7% and 22.1% for resolutions of 42.3–1.68 Å, respectively, with root-mean-square deviations for bond lengths and angles of 0.007 Å and 0.832°, respectively. The Ramachandran plot data showed that 98.74% of the main-chain dihedral angles were in the preferred regions, 0.74% in the allowed regions, and 0.50% in the outlier regions.

**Table 1:** Data collection and refinement statistics. (values in parentheses indicate the highest-resolution shell).

	Native	Hg derivative
Wavelength (Å)	0.9000	1.0070
Space group	*P*4_1_	*P*4_1_
Unit cell parameters (Å)	*a* = *b* = 86.26, *c* = 58.77	*a* = *b* = 85.47, *c* = 58.58
Resolution range (Å)	42.3–1.68 (1.71–1.68)	48.3–2.48 (2.52–2.48)
Total number of reflections	363,111	226,847
Number of unique reflections	48,961 (2,294)	15,172 (722)
Multiplicity	7.4 (7.1)	15.0 (14.4)
*R*_merge_ (%)	4.9 (>100)	6.3 (68.2)
*R*_pim_	2.6 (48.5)	2.0 (20.2)
*CC*_1/2_ highest-resolution shell	0.696	0.907
<*I>*/<σ (*I*)>	25.2 (1.64)	70.7 (4.37)
Completeness (%)	99.3 (94.1)	99.8 (97.0)
**Refinement statistics**		
Resolution range (Å)	42.3–1.68	
Number of reflections	44,320	
*R*_work_ (%)/*R*_free_ (%)	18.65/22.10	
**Root-mean-square deviations**		
Bond lengths (Å)/Bond angles (°)	0.007/0.832	
**Average B-factors (Å** ^**2**^ **)/Number of atoms**		
Protein (Chain A, B)	15.6/1,672, 15.7/1,665	
Small molecules	15.78/24	
Water	29.6/461	
**Ramachandran plot**		
Favored region (%)	98.74	
Allowed region (%)	0.76	
Outliers (%)	0.50	

^a^*R*_merge_ = ∑(*I* - < *I* >)/∑< *I* >, where I is the intensity measurement for a given reflection and < *I* > is the average intensity for multiple measurements of this reflection.

^b^*R*_work_ = ∑|*F*_obs_ - *F*_cal_|/∑*F*_obs_, where *F*_obs_ and *F*_cal_ are the observed and calculated structure-factor amplitudes.

^c^The *R*_free_ value was calculated using only an unrefined, randomly ^c^hosen subset of reflection data (5%) that were excluded from refinement.

^d^Small molecules include acetate ion, and ethylene glycol.

### Structural characteristics of bmGSTu2

**Figure 1:**
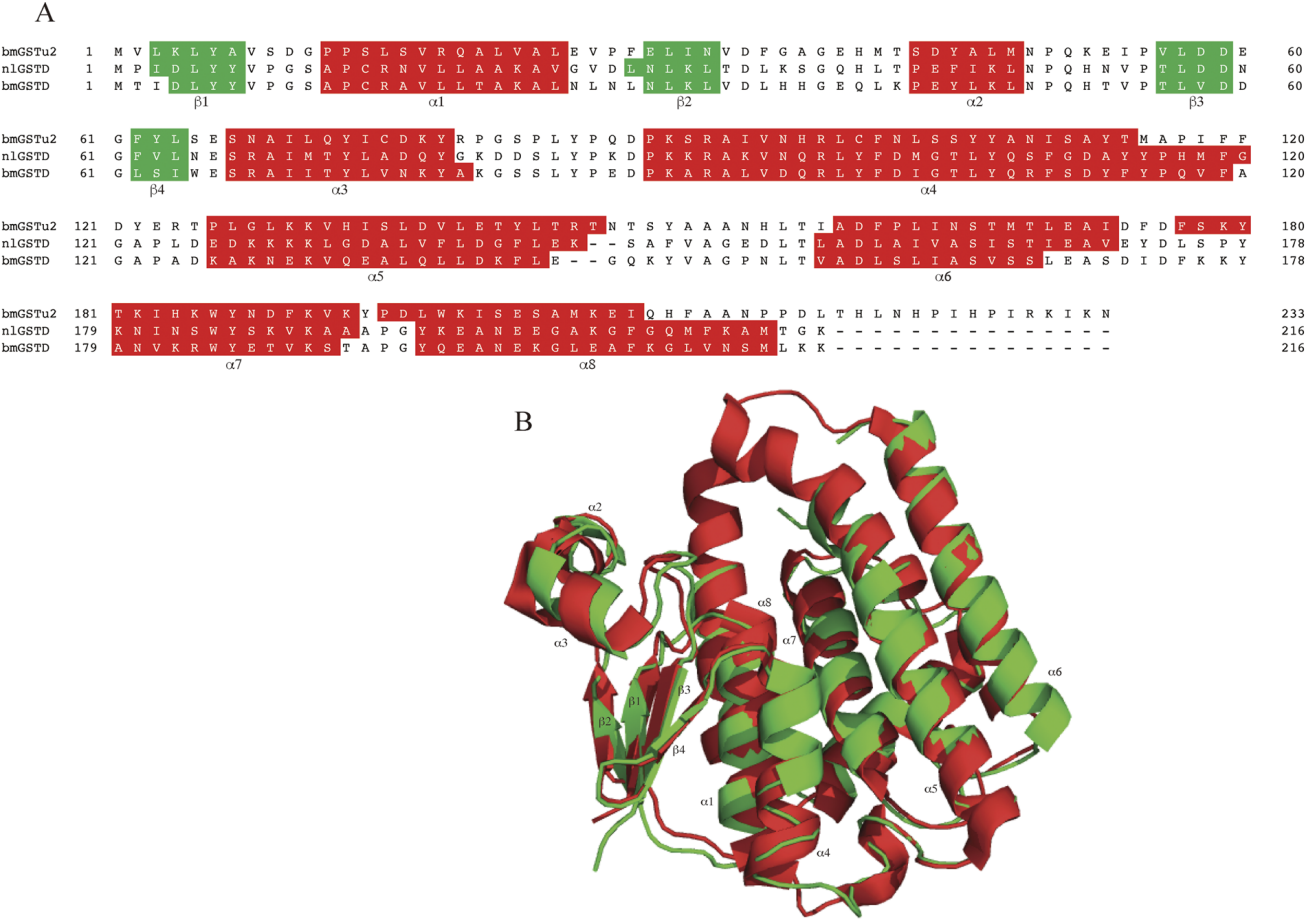
Amino acid sequences of glutathione *S*-transferases (GSTs). Primary alignment and tertiary structure superposition of bmGSTu2 with nlGSTD and bmGSTD. (**A**) α-helices and β-strands are boxed in red and green, respectively. (**B**) Green and red colours indicate bmGSTu2 and nlGSTD, respectively. The starting points of the α-helices and β-strands are shown by α and β, respectively.

The bmGSTu2 amino acid sequence indicated 34% and 33% identity with *N*. *lugens* delta-class GST (nlGSTD, PDB ID: 3WYW) and *B*. *mori* delta-class GST (bmGSTD, PDB ID: 3VK9), respectively (Fig. 1A). The crystal structure of bmGSTu2 was determined at 1.68 Å resolution and solved by the single-wavelength anomalous diffraction (SAD) method using the Hg-derivative. The resulting structure revealed a homodimer of the bmGSTu2 molecule after analysis by PISA program for investigation of macromolecular complexes^19^ and gel filtration elution profile (data not shown). Structural elements, characterised by the STRIDE program for protein secondary structure assignment^20^, showed that bmGSTu2 contains 8 α-helices and 4 β-strands (Fig. 1A). Two discrete domains, N-terminal (residues 1–78) and C-terminal (residues 89–233), were connected by a linker region (residues 79–88) (Fig. 1B). The N-terminal domain included 4 β-strands (β1 [residues 3–7], β2 [residues 29–32], β3 [residues 56–59], and β4 [residues 62–64]) and 3 α-helices (α1 [residues 12–24], α2 [residues 43–48], and α3 [residues 67–78]). The C-terminal domain consisted of α4 (residues 89–114), α5 (residues 126–146), α6 (residues 159–173), α7 (residues 177–193), and α8 (residues 195–208). Similar to other GSTs, the bmGSTu2 structure adopted the canonical GST fold. Screening the predicted 3D model of bmGSTu2 in Protein Data Bank (https://www.rcsb.org) showed highest similarity to nlGSTD (PDB ID: 3WXW) with an E-value of 2.26E-29. The structure of bmGSTu2 reveals root-mean square-deviation of 1.50 Å to that of nlGSTD.

### Amino acid residues crucial for enzymatic function

**Figure 2:**
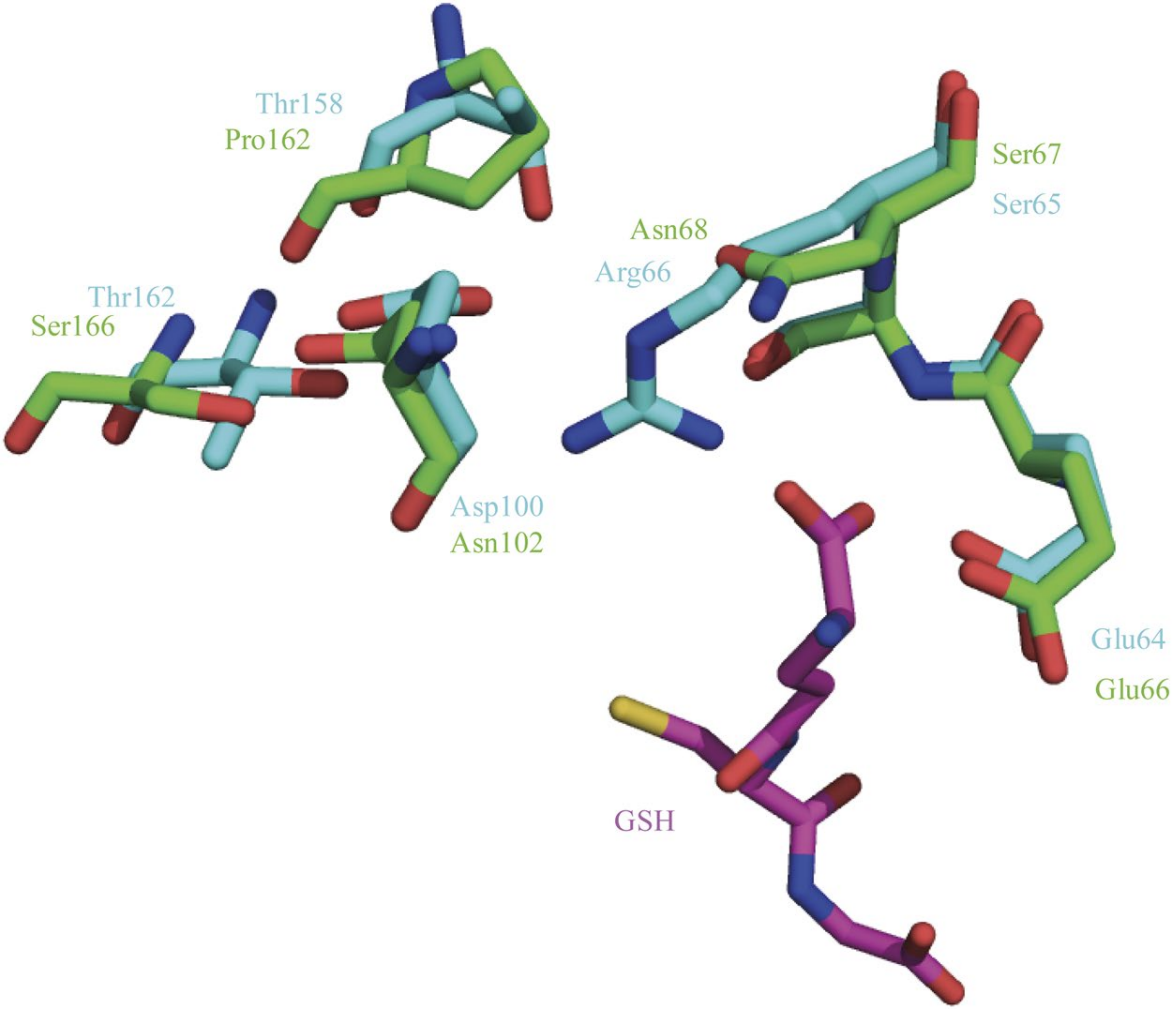
Amino acid residues of the electron-sharing network. Carbon atoms for bmGSTu2, agGSTD3-3, and GSH are represented by green, cyan, and magenta, respectively. Atoms of oxygen, nitrogen, and sulphur are red, blue, and yellow, respectively. Amino acid residues for bmGSTu2 and agGSTd1-6 are described in green and cyan, respectively.

The GSH-binding site (G-site) and substrate-binding site (H-site) include amino acid residues important for enzymatic activity. Previously, we examined G-site components for bmGSTu2^18^. For GSH activation, an electron-sharing network was proposed for a delta-class GST in *Anopheles dirus* (adGSTD3–3), and the network includes Glu64, Ser65, Arg66, Asp100, Thr158, and Thr162^21,22^. The corresponding residues in bmGSTu2 are Glu66, Ser67, Asn68, Asn102, Pro162, and Ser166 (Fig. 2).

H-site variations influence GST substrate specificity. To determine which GST structure is suitable for H-site analysis, a Dali search (http://ekhidna2.biocenter.helsinki.fi/dali/) employing the crystal structure of bmGSTu2 was used to find enzymes showing the highest structural homology to bmGSTu2. Among the GSTs, the structure of delta-class *B*. *mori* (bmGSTD) GSTs was the most similar, with root mean square deviations of 1.4 Å. For the modelled bmGSTu2 structure, delta-class GSTs consistently showed the greatest homology^18^. The putative bmGSTD H-site (PDB ID: 1PN9) contains Leu5, Ala12, Pro13, Leu35, Tyr107, Phe110, Tyr113, Phe119, and Phe206. In the bmGSTu2 amino acid sequence, 5 of the 12 residues (Pro13, Tyr107, Ile118, Phe119, and Phe211) are identical to those in bmGSTD.

**Figure 3:**
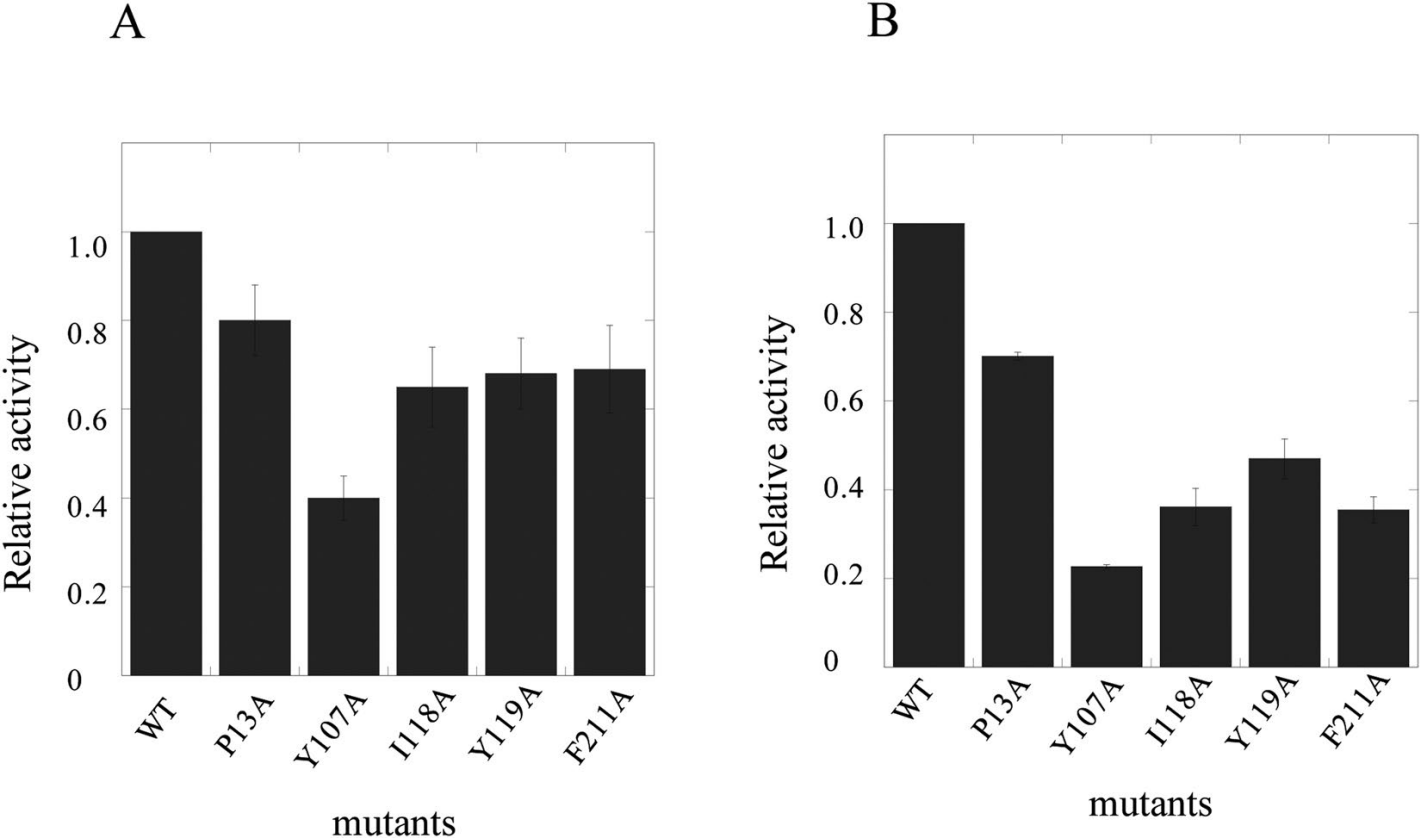
Specific activities of bmGSTu2 mutants in reactions with CDNB (**A**) and diazinon (**B**). The activities of wild-type (WT) and mutants (P13A, Y10A, I118A, Y119A, and F211A) are shown. Data represent the mean values of three independent experiments. Statistics were performed using one-way ANNOVA. Significant level is at P < 0.05.

In the bmGSTu2 structural model, the electron densities from the following regions were poor for modelling: Ala116 to Glu123, after Phe211 of the A chain, and from Pro117 to Tyr122. On the B chain of bmGSTu2, the electron densities from Ala116 to Glu123, after Gln209 of the A chain, and from Pro117 to Tyr122 were also poor for modelling. To examine whether Pro13, Tyr107, Ile118, Phe119, and Phe211 contribute to bmGSTu2 activity, we mutated these amino acid residues to Ala. The resulting mutants were named P13A, Y107A, I118A, F119A, and F211A. After purification from *Escherichia coli*, we detected a single band in each final preparation upon SDS-PAGE analysis (data not shown). The specific activities of bmGSTu2 mutants were compared to those of the wild-type enzyme toward CDNB (Fig. 3A) and diazinon (Fig. 3B). For both the substrates, the activities of the mutants were decreased. The Y107A mutant resulted in the most prominent decrease in activity among all the mutants tested to date.

### Establishment of the mutant allele for *bmgstu2*

**Figure 4:**
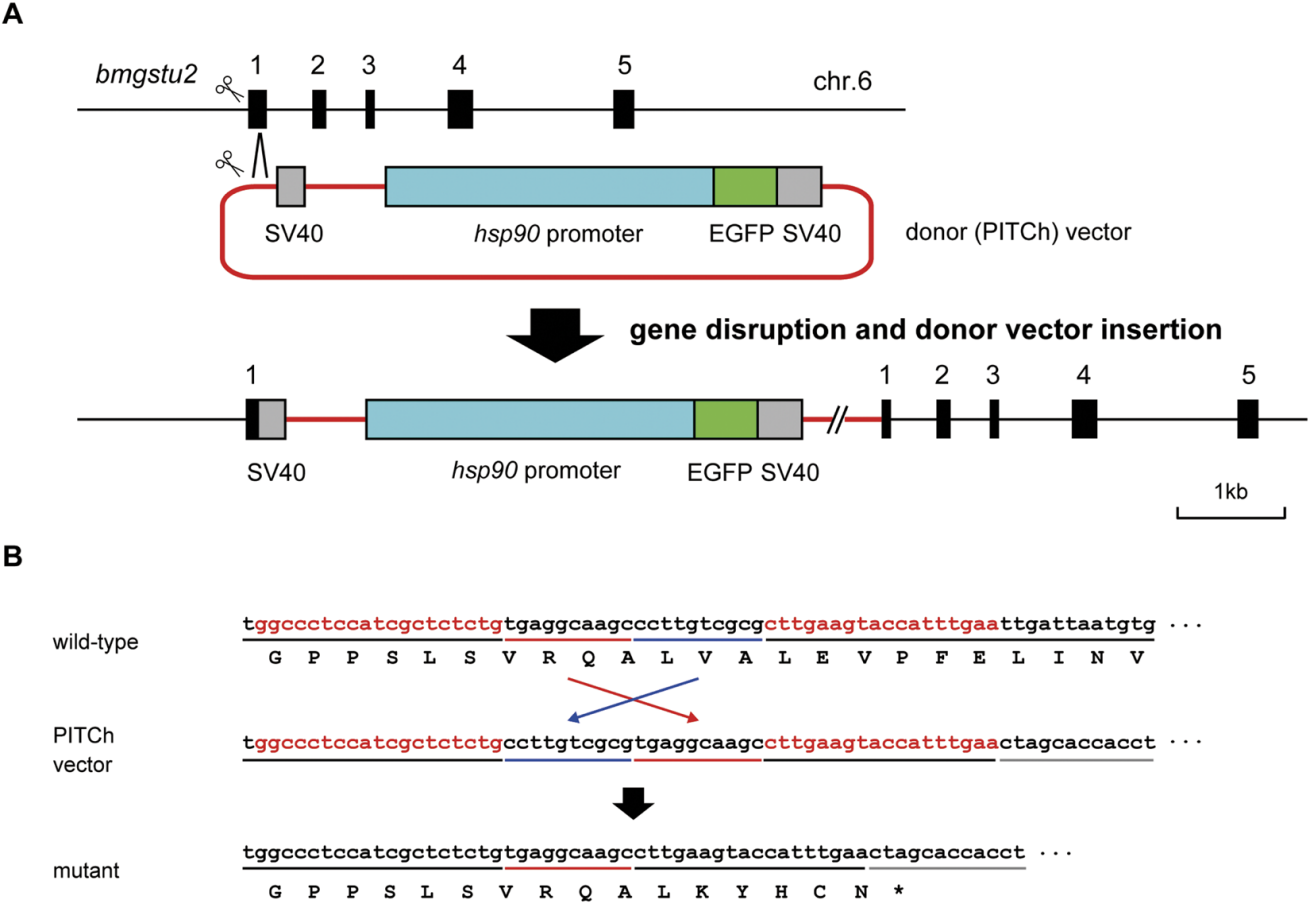
Establishment of the mutant strain. (**A**) Schematic representation of *bmgstu2* mutant allele. The structure of the *bmgstu2* gene is shown at the top. Black bars indicate exons of *bmgstu2*. The scissors indicate TALENs that target both the genome and PITCh vector including SV40, the *hsp90* promoter, and EGFP. (**B**) The resulting sequence of the mutant allele. The red character indicates the TALEN target site. The black underline represents the sequence derived from *bmgstu2*; red and blue lines represent microhomology sequences, and the grey line indicates a partial sequence of SV40. The precise donor (PITCh) vector insertion was present in the established strain.

We established a mutant *bmgstu2* silkworm strain to better determine its function (Fig. 4). To create this mutant strain, we used TALEN, which is a promising genome editing tool to disrupt the target gene efficiently in the silkworm and other organisms^23^. This approach involves integration of the donor plasmid into the target genome locus using the TAL-PITCh method, a TALEN-based knock-in system^24,25^. This enables the discrimination of the wild-type and mutant allele easily. In the present study, we disrupted the *bmgstu2* gene and inserted the GFP sequence as a reporter (Fig. 4). TALEN vectors designed against the coding region of the *bmgstu2* gene were microinjected into 300 silkworm embryos using a mixture of the donor vector (PITCh vector), resulting in a number of GFP-positive G1 individuals. The genotyping analysis revealed that 15 individuals showed targeted gene disruption (Fig. S1). The sequencing analysis revealed that one of these individuals showed precise integration for both 5′ and 3′ junctions (data not shown), and we utilised this mutant strain for further functional analysis of *bmgstu2*.

### Median lethal dose (LD50) and acetylcholine (ACh) measurements

**Figure 5:**
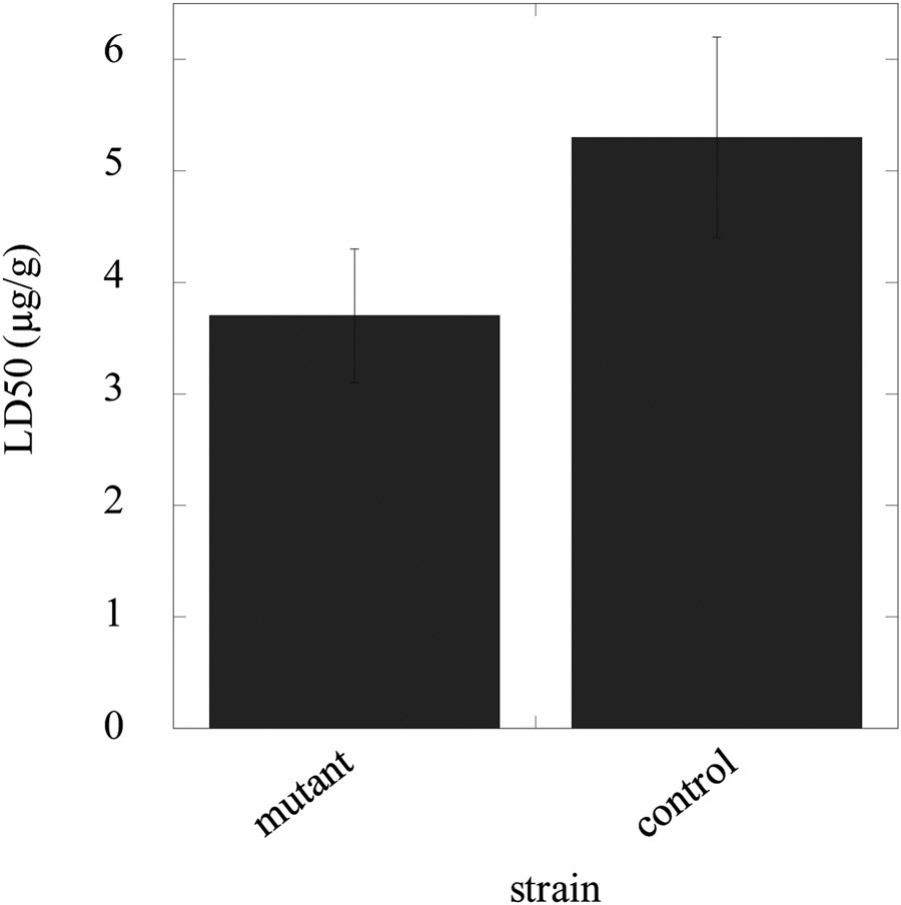
The effect of diazinon on LD50 values. Day-1 fifth-instar larvae of the mutant (homozygote) and control (heterozygote) were exposed to diazinon solutions via direct contact with the larval abdomen. At 24 h post-treatment (on day 2), LD50 values were determined. Relationships between two variables were examined by one-way ANOVA, with a significance level at P < 0.05.

**Figure 6:**
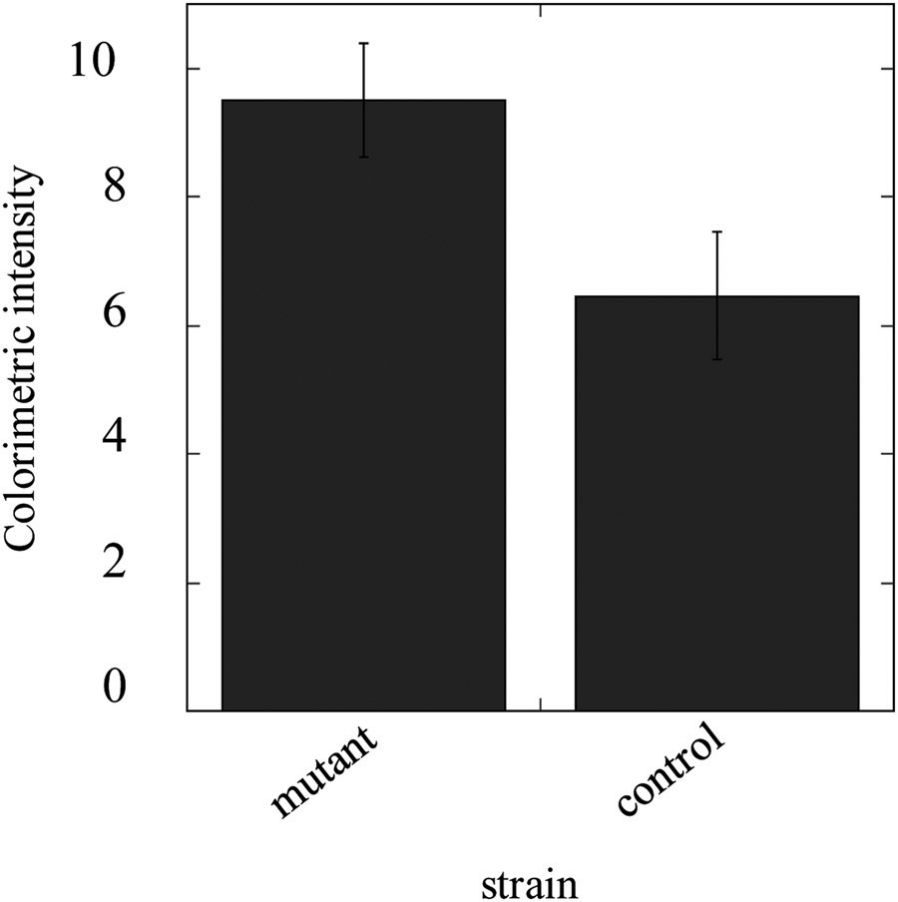
The effect of diazinon on ACh content. Crude extracts were prepared from the whole bodies of mutant (homozygote) and control (heterozygote). ACh production was determined by a colorimetric assay described in the Methods section. The colorimetric intensity was normalised to body weight, in grams. The intensities are expressed as the means of triplicate experiments. The significance of differences between each group was calculated based on one-way ANOVA analysis (P < 0.05).

We measured the LD50 levels for diazinon, a widely used organophosphate insecticide, in the mutant and control *B*. *mori* strains. The LD50 value in the mutant was decreased to 70% of that in the control strain of *B*. *mori* (Fig. 5). Acetylcholine (ACh) levels were also estimated by using acetylcholinesterase (AChE) and choline oxidase. Binding of organophosphate insecticide to AChE results in inactivation of AChE, which indicates that AChE is unable to metabolise ACh. Organophosphate insecticides exert their toxicity by allowing ACh to overact at its receptors in the central and peripheral nervous systems. Notably, ACh levels in the control strain were decreased to 70% of that in the knock-in strain (Fig. 6).

## Discussion

In our previous study, we identified and characterised bmGSTu2^18^. We also found that bmGSTu2 was capable of metabolising diazinon. Mutation of putative amino acid residues in the G-site showed that Ile54, Glu66, Ser67, and Asn68 are crucial for enzymatic function.

In the present study, we focused on the crystal structure of bmGSTu2 to examine amino acid residues that contribute to the conjugation reaction of diazinon on GSH, as well as the physiological role of bmGSTu2 by genome editing analysis using TALEN. Additionally, we solved the crystal structure of bmGSTu2. Superposition of the structure of bmGSTu2 onto that of nlGSTD demonstrated that the α-helices and β-strands of bmGSTu2 were conserved across the structures (Fig. 1A,B).

Although amino acid residues in the G-site are conserved in *B*. *mori* GSTs^11,26–30^, the sequence diversity of the H-site is attributed to substrate specificity^31^; furthermore, this diversity may be the reason why substrate specificities of *B*. *mori* GSTs are varied. Our mutagenesis experiments indicate that the residues Pro13, Tyr107, Ile118, Phe119, and Phe211 in bmGSTu2 play important roles in its enzymatic functions. Among all the mutants tested, the Y107A mutation showed the most apparent decrease in activity. Another critical residue, Tyr107, is located in the H-site and is highly conserved among delta-class GSTs, whereas the Phe residue is conserved instead of Tyr among epsilon-class GSTs^32^. Similar results were obtained for *A*. *dirus* delta-class GST4-4 (adGST4-4). Tyr111 of adGSTD4–4, the corresponding residue to Tyr107 of bmGSTu2, was reported to contribute to substrate-binding and stabilisation of GSH^33^. Among the GSTs, the H-site of delta-class *A*. *gambiae* GSTs (adGSTd1-6) was the most similar. The H-site of agGSTd1–6 (PDB ID: 1PN9) is composed of residues that are mostly hydrophobic: Leu6, Ala10, Pro11, Leu33, Met34, Tyr105, Phe108, Tyr113, Ile116, Phe117, Phe203, and Phe207^34^. In the bmGSTu2 sequence, 5 of the 12 residues (Pro13, Tyr107, Ile118, Phe119, and Phe211) are equivalent to the above agGST1-6 residues, and the remainder (Val8, Phe35, and Ile110) exhibit similarity to those in agGSTd1-6.

Other sites proposed to be requisite for bmGSTu2 function are the lock-and-key motif, the small hydrophobic core, and an ionic bridge^21,22,35,36^. The lock-and-key motif is important for stabilising hydrophobic interactions of GST monomers^22,27^. An intersubunit motif (lock-and-key) was found in the dimer interface of bmGSTD, a delta-class GST^27^, wherein the ‘key’ residues (Glu66, Arg68, and Tyr100) are introduced into a hydrophobic region (Ala69, Leu99, Tyr100, and Ile103), the ‘lock’ of the neighbouring subunit. Given that bmGSTu2 contains Glu66, Asn68, Ala69, Leu99, Cys100, and Lue103 in its amino acid sequence, it may potentially conserve the lock-and-key system. A small hydrophobic core and an ionic bridge (Leu6, Thr31, Leu33, Ala35, Glu37, Lys40, and Glu42) contributing to the stabilisation of the α helix were reported in adGSTD4-4^33^. The corresponding residues in bmGSTu2 are Val8, Val33, Phe35, Ala37, Glu39, Thr42, and Asp44.

The electron-sharing network can be divided into type I and II classes^21,22^. The type I electron-sharing network is exemplified by delta-, theta-, omega-, and tau-class GSTs, which have an acidic amino acid residue at position 64, whereas type II network GSTs (alpha, mu, and pi, and sigma classes) contain a polar amino acid residue (glutamate) at this site that is capable of interacting with the γ-glutamyl portion of GSH. We demonstrated that Gln66 is conserved in the bmGSTu2 sequence, which is a characteristic of type II networks.

To determine the physiological role of bmGSTu2 in *B*. *mori*, we successfully constructed a mutant of this gene using a genome-editing approach. We then exposed silkworm larva to diazinon and measured the resulting LD50 values and ACh levels *in vivo*. The decreased LD50 value observed for the mutant after diazinon exposure indicates that bmGSTu2 is involved in diazinon tolerance *in vivo*. The organophosphate insecticide diazinon is a specific inhibitor of AChE, which is a common neurotoxicity biomarker. Once diazinon binds to AChE irreversibly, AChE is unable to metabolise ACh. We found that the control strain preferentially contains ACh, compared to that in the mutant. This result indicates that the reduced ACh levels in the mutant silkworm strains after diazinon exposure did not occur through AChE inhibition. However, we did not observe a complete disappearance of ACh in the knock-in strain. Thus, there might be other detoxification enzymes responsible for diazinon degradation. This may also be the reason why the LD50 value was not decreased to more than 70%. Cytochrome P450 and esterase, for example, are major detoxification enzymes that are able to degrade organophosphorus insecticides^37^. The silkworm genome reveals the existence of these enzymes in *B*. *mori*. To understand their involvement in the insecticide detoxification system in this species, it may be useful to compare their detailed properties, such as expression rates, activities, substrate specificities, and resistance spectra. Investigations along these lines are underway in our laboratories.

In summary, we provide evidence that bmGSTu2 contributes to diazinon tolerance in *B*. *mori*. We identified amino acid residues of bmGSTu2 that play important roles in catalysis. We are currently attempting co-crystallisation of bmGSTu2 with diazinon or a suitable substrate analogue conjugate to aid in the determination of amino acid residues involved in bmGSTu2 catalysis. The existence of a bmGSTu2 homologue in other agricultural pests must be determined to understand their importance for other insecticide detoxification system. Together, our findings may facilitate the development of more effective and safe insecticides.

## Methods

### Crystallisation and preparation of heavy atom derivative

Recombinant bmGSTu2 was purified as described previously^38^ and then prepared using a centrifugal filter (Merck, Darmstadt, Germany) to 10 mg/mL in 20 mM Tris-HCl buffer, pH 8.0, containing 0.2 M NaCl. Crystallisation drops were formed by mixing an equal volume (1 μl) of protein and reservoir solutions for a hanging drop vapour diffusion method. Native crystals were grown at 20 °C for a week in 0.1 M Tris-HCl pH 8.0 containing 0.6 M sodium acetate and 25% PEG4000. Crystals of the mercury derivative were obtained by soaking native crystals in 0.1 M Tris-HCl pH 8.0 containing 0.6 M sodium acetate, 25% PEG4000, and 5 mM chloromethylmercury for 5 h at 20 °C.

### Data collection and structural determination

For data collection, crystals were selected using a cryoloop and flash frozen with liquid nitrogen. X-ray diffraction data collections were performed using synchrotron radiation on a SPring-8 beamline BL44XU^39^ with *λ* = 0.9000 Å for the native data set and with *λ* = 1.0070 Å (Hg peak) for the heavy atom data set, in a nitrogen vapour stream at 100 K. Data sets were integrated and scaled using the program *DENZO* and *SCALEPACK* as implemented in the *HKL2000* program package^40^. Phasing was performed by the single-wavelength anomalous diffraction (SAD) method using the Hg-derivative in the program *SHELX C*, *D*, and *E*^41^ as implemented in *HKL2MAP*^42^; the initial model was constructed using the program *ARP/wARP*^43^. The model constructed using the Hg-derivative data was used as a search model for a molecular replacement method with the program *Phenix Phaser-MR*^44^ against the native data. The program *ARP/wARP* was used for further model building. After manual adjustment using the program *Coot*^45^, refinement was carried out using the program *phenix*.*refine*^46^.

### Site-directed mutagenesis

Site-directed mutagenesis of bmGSTu2 were performed using the Quick-Change Site-Directed Mutagenesis kit (Stratagene Corp., La Jolla, CA), based on the manufacturer’s instructions. The mutagenesis primers were as follows: 5′- ATCGGAATTAATTCGGATCCGAATTCATGGTTCTAAAATTATATGCCGTTTCTGATGGCCCTGCTTCGCTCTCTGTGAGGCAAGCC −3′ for P13A, 5′-GTGTAGGCTGAGATATTAGCAGCATAGCTTGATAAGTTAAAAC-3′ and 5′-GTTTTAACTTATCAAGCTATGCTGCTAATATCTCAGCCTACAC-3′ (antisense) for Y107A, 5′-GTGCGTTCGTAGTCAAAGAAAGCAGGTGCCATTGTGTAGGCTG-3′ and 5′-CAGCCTACACAATGGCACCTGCTTTCTTTGACTACGAACGCAC-3′ for I118A, 5′-TGGAGTGCGTTCGTAGTCAAAAGCTATAGGTGCCATTGTGTAGG-3′ and 5′-CCTACACAATGGCACCTATAGCTTTTGACTACGAACGCACTCCA-3′ for F119A, and 5′-ATAGGGTGTATAGGATGGTTGAGGTGAGTCAAGTCTGGAGGGTTAGCGGCAGCATGTTGGATCTCTTTCATGG-3′ and 5′-ATCTCAGTGGTGGTGGTGGTGGTGCTCGAGTTAATTTTTGATTTTTCGGATAGGGTGTATAGGATGGTT-3′ for F211A. An expression plasmid for preparation of the recombinant bmgstu2 was used as a template, and each mutagenesis was confirmed by DNA sequencing of full-length mutated cDNA.

### Measurements of enzyme activity

GST was assayed spectrophotometrically using 1-chloro-2,4-dinitrobenzene (CDNB) and 5 mM GSH as standard substrates^47^. GST activity was expressed as mol CDNB conjugated with GSH per min per mg of protein. The ability of bmGSTu2 to metabolise diazinon was determined by HPLC according to previous reports. The eluate was monitored at 246 nm for the detection of metabolites. Specific activity toward diazinon was determined on the basis of the corresponding peak area identified per mg of protein.

### Construction of TALEN and PITCh vectors

TALEN vectors were constructed as described by Takasu *et al*. (2014, 2016)^48,49^. The target site was selected within the coding region of the *bmgstu2* gene, and the sequence around the target site was determined in the *w1*-*pnd* strain. TALEN was assembled using the Golden Gate TALEN and TAL Effector kit (Addgene, Cambridge, USA) and the TALEN backbone vector pBlue-TAL^23^. The mRNA was *in vitro* synthesised using the mMESSAGE mMACHINE T7 kit (Ambion, Carlsbad, USA).

PITCh vector construction was carried out following the method described in Tsubota and Sezutsu (2017)^50^. Inverse PCR was carried out using the primers 5′-TGAGGCAAGCCTTGAAGTACCATTTGAACTAGCACCACCTGTTCCTGTAG-3′ and 5′-CGCGACAAGGCAGAGAGCGATGGAGGGCCACTCGAATTAGATCTTTGG-3′ against the pBachsp90GFP-3xP3DsRed plasmid^24,51^. The PCR product was self-ligated, and the inserted sequence was checked using Applied Biosystems 3130*xl* (Life Technologies) after cycle sequencing with BigDye Terminator V3.1 (Life Technologies, Carlsbad, USA).

### Microinjection

Microinjection was carried out following the method described in Tamura *et al*.^52^. The TALEN mRNAs and PITCh vector were injected into *w1*-*pnd* embryos at the syncytial preblastderm stage. The TALEN mRNA concentration was 125 ng/µL each, and the PITCh vector concentration was 500 ng/µL.

### Genotyping of the mutant individuals

Genomic DNA was extracted using the DNeasy kit (QIAGEN, Hilden, Germany) for each G1 adult from the crosses. PCR amplification was carried out using primers 5′-CACGAGTACAGAGAATATGG-3′ and 5′-ATTTGTTGGCAGCACTGCTT-3′ for the 5′ junction and 5′-ATAACGACCGCGTGAGTCAA-3′ and 5′-GGTAGTACTCGTTAGCTAGC-3′ for the 3′ junction. The amplicons were sequenced using Applied Biosystems 3130*xl* after cycle sequencing with BigDye Terminator V3.1.

### Tissue dissection

Each tissue was isolated on ice from day-3 fifth-instar larvae and kept at –80 °C until use. Day-1 fifth-instar larvae were exposed to various concentrations of diazinon solutions via direct contact with the larval abdomen. At 24 h post-treatment (on day 2), LD50 values were recorded. For genome-editing experiments, the *w1*-*pnd* (non-diapausing) strain was used to establish the mutant strains. The established strains were crossed with the *w*-*c* (diapausing) strain to maintain the stocks. These strains were reared using an artificial diet (Nihon Nosan Kogyo, Yokohama, Japan) at 25 °C under a 12-h light/dark photoperiod.

### Acetylcholine (ACh) measurements

The ACh content of silkworms was measured using a colorimetric acetylcholine assay kit (Cell Biolabs Inc., San Diego, CA, USA). Briefly, whole bodies of the silkworms were homogenised in chloroform/methanol (2:1, v/v). After centrifugation, the lower phase was collected and dried completely. The resulting extract was dissolved in chloroform/methanol/water (86:14:1, v/v/v) and used as crude extract. Acetylcholinesterase and choline oxidase were added to detect acetylcholine. Acetylcholine content was measured at a wavelength of 540 nm after incubation and estimated as the arbitrary colorimetric units normalised to the milligrams of body weight used.

## Electronic supplementary material

Figure S1
