# Supplementary figures and images for: Characterisation of a diazinon-metabolising glutathione S-transferase in the silkworm Bombyx mori by X-ray crystallography and genome editing analysis
Source: Sci Rep. 2018 Nov 15;8:16835. doi: 10.1038/s41598-018-35207-8 (PMC6237972; doi:10.1038/s41598-018-35207-8)

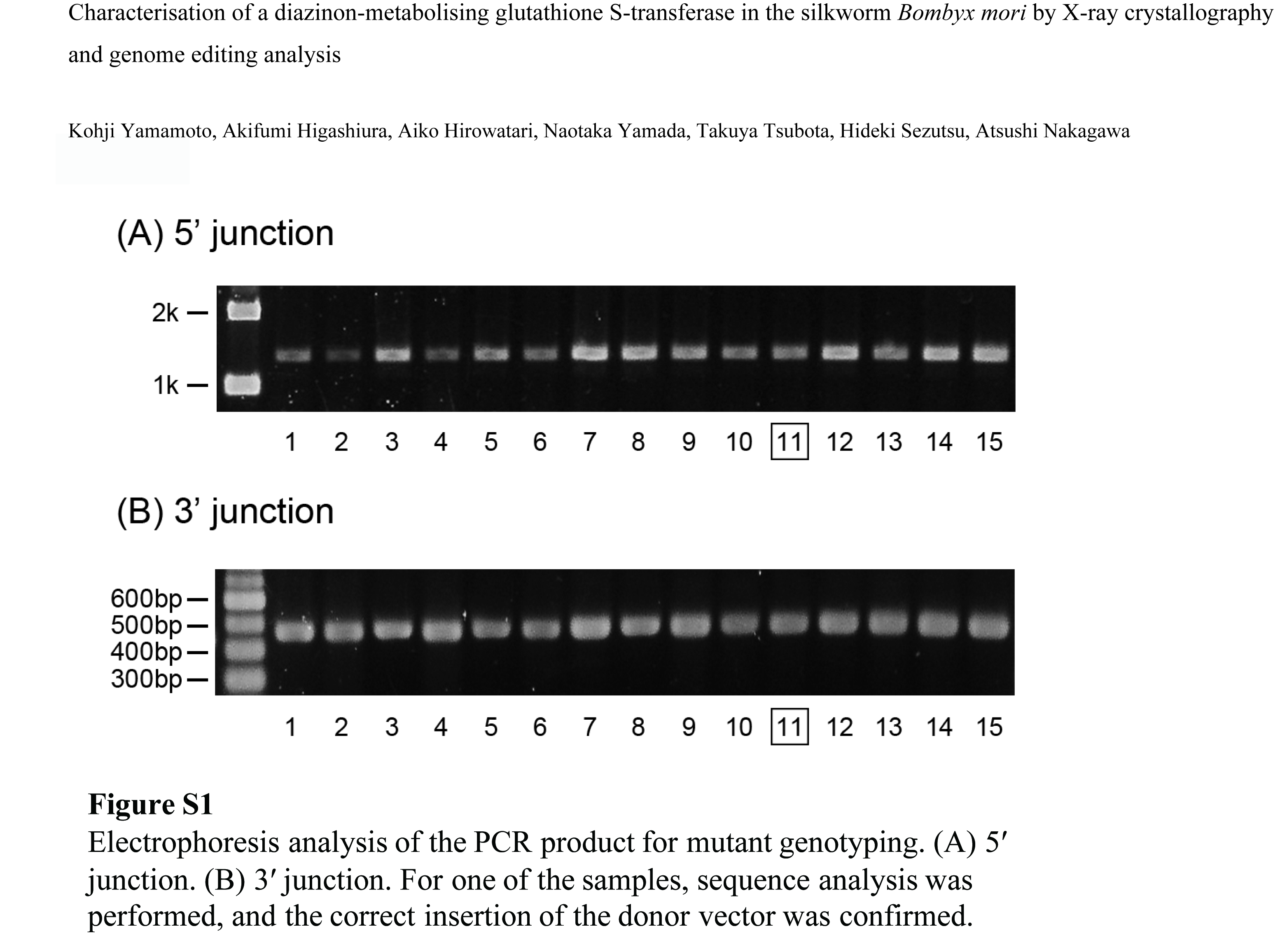

Supplement: Supplementary file 1 — Figure S1 [file 41598_2018_35207_MOESM1_ESM.tif]
